# Waist-to-Height Ratio Is More Predictive of Years of Life Lost than Body Mass Index

**DOI:** 10.1371/journal.pone.0103483

**Published:** 2014-09-08

**Authors:** Margaret Ashwell, Les Mayhew, Jon Richardson, Ben Rickayzen

**Affiliations:** 1 Ashwell Associates, Ashwell, UK and Visiting Research Fellow, Oxford Brookes University, Oxfordshire, United Kingdom; 2 Cass Business School, City University London, Faculty of Actuarial Science and Insurance, London, United Kingdom; University of Alabama at Birmingham, United States of America

## Abstract

**Objective:**

Our aim was to compare the effect of central obesity (measured by waist-to-height ratio, WHtR) and total obesity (measured by body mass index, BMI) on life expectancy expressed as years of life lost (YLL), using data on British adults.

**Methods:**

A Cox proportional hazards model was applied to data from the prospective Health and Lifestyle Survey (HALS) and the cross sectional Health Survey for England (HSE). The number of years of life lost (YLL) at three ages (30, 50, 70 years) was found by comparing the life expectancies of obese lives with those of lives at optimum levels of BMI and WHtR.

**Results:**

Mortality risk associated with BMI in the British HALS survey was similar to that found in US studies. However, WHtR was a better predictor of mortality risk. For the first time, YLL have been quantified for different values of WHtR. This has been done for both sexes separately and for three representative ages.

**Conclusion:**

This study supports the simple message “Keep your waist circumference to less than half your height”. The use of WHtR in public health screening, with appropriate action, could help add years to life.

## Introduction

Obesity is a condition used to describe high levels of body fat and is associated with increased risk of morbidity and mortality. The measure of obesity most commonly used is the Body Mass Index (BMI), defined as weight/height^2^. Individuals are classified as overweight if their BMI is between 25 kg/m^2^ and 30 kg/m^2^, obese if it is between 30 and 40 kg/m^2^ and morbidly obese if it is greater than 40 kg/m2. Using BMI to measure obesity, the Health Survey for England (HSE) shows that the proportion of adults classed as obese has increased in the UK from 15% in 1993 to 26% in 2010 [1]. Obesity in men and women is at its highest level since records began, with 26.2% men and 26.1% women classified as obese [2].

In recent years, indices of central obesity (first waist-hip ratio “WHpR” and then waist circumference “WC”) have increasingly been associated with higher cardiometabolic risk than BMI in both cross-sectional and prospective studies. The use of waist-to-height ratio (WHtR) for detecting central obesity, and health risks associated with it, was first proposed in the mid 1990s by one of the authors and others [3] [4] [5]. Using regression analysis on ten-year follow-up Health and Lifestyle Survey (HALS) data, Cox and Whichelow found that BMI was not a significant predictor of death from all causes. By contrast, they found that WHtR was significant in predicting all-cause mortality [6]. Since then interest in the effectiveness of this measure has risen in both adults and children in many different ethnic groups and countries.

A recent meta-analysis [7] concluded that robust statistical evidence from studies, involving more than 300, 000 adults in several ethnic groups, showed the superiority of WHtR over WC and BMI for detecting cardiometabolic risk factors in both sexes. Most studies support a boundary value (a preferred term to ‘cut-off value’) for minimal risk of WHtR of 0.5 [8] and this value is being rapidly adopted in many studies [9]. Given that WHtR is a better risk measure than both BMI and WC, but that BMI has been the traditional measure of obesity, we focused our analysis on WHtR and BMI (ie we did not include WC in our study).

The UK government is concerned that the cost of obesity will be felt by every single part of society, not just in headline financial or health terms but in very personal ways, describing obesity as the equivalent of the ;climate change’ of public health [9]. The reasons for this public and government concern are clear to see. Obesity is associated with an increased risk of various life threatening diseases such as cancer, cardio-vascular diseases and diabetes [10], [11]. For example, an obese woman, compared with a healthy weight woman, is almost thirteen times more likely to develop type 2 diabetes [12]. These diseases lead to reduced life expectancy. The most recent UK Government policy document [13] outlines a call to action for just this reason. Studies into the relationship between BMI and mortality have found that the risk of death increases when BMI is less than 20 kg/m^2^, is minimal between 20 kg/m^2^ and 25 kg/m^2^ and increases for BMI categories above this level [14] [15].

Much of the research into the effects of obesity on life expectancy has been done using BMI as the measure of obesity and has focused on the US population [21]. In this paper we argue that, based on the evidence, WHtR is a better measure of obesity to use and, for the first time, we are able to quantify the years of life lost (YLL) due to obesity as measured by WHtR. We also quantify the YLL through being obese as measured by BMI in order to be able to compare our results (which are based on the British population) with previous studies using BMI which are based on the US population. We find that the results using BMI are consistent between the two countries.

As mentioned above, we contend that WHtR is a better measure of the health risks due to obesity than BMI. BMI overestimates fat in muscular people and cannot give information about fat distribution. In contrast, WHtR is a better proxy for central fat, which has greater associated health risks than fat stored in other parts of the body.

To bring these strands together, the aim of the research was to estimate YLL in an obese individual where obesity was measured either by BMI or WHtR. We calculated the effects of obesity on life expectancy at representative ages and for each gender separately. We found that central obesity, as measured by WHtR, was a better predictor of mortality and YLL than BMI. We conclude that, compared with BMI, WHtR is more valuable for health screening purposes, for delivering public health policies and for estimating the burden of obesity on society.

## Methods

### Sources of information

YLL is defined as the difference in life expectancy between an individual whose anthropometric indices are optimally healthy and an individual with a sub-optimal measurement. To calculate YLL, we combined information from three sources: the Health Survey of England [16] the Health and Lifestyle Survey 1985 [17] and 2006 UK interim life tables [18], [19].

The Health Survey for England (HSE) provides cross-sectional information on the prevalence of obesity in the population by age and gender. This survey is conducted annually for the National Health Service (NHS) in order to monitor the nation’s health through surveying the population for specified health issues [20].

The Health and Lifestyle Survey (HALS) is a longitudinal study of health and behaviour based on a representative random sample of the British population (England, Wales and Scotland). It was initiated in 1985 and now contains information on more than 20 years of follow-up data on participants who have died [17]. HALS is similar to the National Health and Nutrition Survey (NHANES) used for the US population, so the methodology used in the US study [21] is replicated in this paper, allowing direct comparison of results between Britain and the US.

HALS provides a long term picture of the British population’s health and physiological characteristics including the following attributes: weight, height and waist measurements, smoking behaviour, alcohol consumption, diet and physical exercise; associated health implications; beliefs and perceptions about health and lifestyle; relationships between lifestyle and physiological status and the effect of cognitive ability on these issues.

HALS data were collected by interview. Physiological information, including measures of obesity, was collected through a nurse visit. The dataset included 7,414 respondents from the age of 18. Almost the entire sample is linked with the NHS central register allowing death information for these respondents to be updated periodically. Our results are based on the survey results up to 2005. Nearly 2000 deaths have been recorded as shown in Table 1. It should be noted that, since our work was carried out, the survey results have been updated to 2009.

**Table 1:** HALS population samples and number of deaths.

Measure	Smoking Status	Male Subjects	Male Deaths	Female Subjects	Female Deaths
BMI	Non-smokers	1,685	473	2,609	608
BMI	All	3,297	952	3,979	893
WHtR	Non-smokers	1,678	472	2,597	603
WHtR	All	3,281	949	3,958	887

### Overall strategy and calculation of years of life lost (YLL)

Our strategy was to identify the pure effects of obesity on years of life lost (YLL) by excluding smokers from our analyses. Smoking is a competing cause of death. Therefore, by excluding smokers, we removed any distortion in our YLL results which would come from the risks associated with smoking, rather than those from obesity. Although it is possible to carry out a similar analysis for smokers, it is difficult to present a clear set of conclusions since, apart from the competing risk point, the amount that smokers smoke (ie whether an individual is a habitual heavy or light smoker) is not captured as accurately as we would wish and so will affect the results.

For a comparison of the relative impact which obesity and smoking has on life expectancy, the reader is referred to a recent review [22]. The authors note that, certainly in the US (and this is particularly true of the UK), the proportion of the population that smokes has been decreasing over a long period. It is hence arguably more pertinent that we concentrate on non-smokers in this study.

There were four steps in the calculation of YLL:

1. An estimate of the distribution of anthropometric indices of interest (BMI and WHtR) within the total population was made for each year of adult life from age 18 to 85 years using data from HSE published in 2006.
2. Using HALS data, estimates of the Cox proportional hazards ratio for death, based on BMI and WHtR values and age, were obtained. This was done by investigating the association between the obesity of participants at the start of the study and their subsequent mortality. The role of the Cox model is to identify the relationships between risk factors that affect a subject’s survival.
3. For each age, the relative risks associated with different levels of obesity were combined with the distribution of obesity in the population. This was then used to decompose the population life table into ‘impaired life’ tables for obese groups.
4. Using the resulting ‘impaired life’ tables, the mean life expectancy for each age was derived and compared with the corresponding figure from the non-obese life table to calculate YLL. The mean was then used to allow direct comparison with previous research [21] [23]. The population life table used was the 2006 interim life table for the United Kingdom, produced by the Office for National Statistics [18].

### Analysing the distribution of anthropometric indices

Using the HSE data, the proportion of the population in the following BMI categories were estimated: under 17 kg/m^2^, 17 to <18, 18 to <19, 19 to 20 etc up to 44 to <45, and over 45 kg/m^2^. The categories for WHtR were: under 0.36, 0.36 to <0.38, 0.38 to <0.40 etc up to 0.78 to <0.80, and over 0.80.

The BMI and WHtR data was smoothed to remove distortions across ages caused by sample error. We adopted the same smoothing procedure as used in [21] on the HSE data. The probability of being in the following 32 overlapping BMI categories was estimated: 13 kg/m^2^ to <18 kg/m^2^, 14 to <19, 15 to <20 up to 44 to <49. The categories for WHtR were: 0.28 to <0.36, 0.30 to <0.38, 0.32 to<0.40 etc up to 0.80 to <0.88.

Researchers using US data [21] [24] found that a third degree polynomial accurately characterised the convex relationship between change in age and BMI. Since we found no contradictory evidence from Britain, the same approach was followed for BMI and WHtR in this study. The probability of being in each interval was estimated for each age from 18 to 85 years using the resulting equations. Then, within each age, the probability of being in each one unit interval was estimated as the moving average of the wider intervals containing the one unit interval. For example, the percentage of the population for BMI of 18 would be the average of BMI 14 to <19, 15 to <20, 16 to <21, 17 to <22 and 18<23.

A smoothed distribution of BMI and WHtR for ages 18 to 85 years was obtained. Having carried out this procedure, we verified that the smoothed total population obesity distribution was very close to the unsmoothed distribution. The mean values of BMI and WHtR for each interval were then calculated from the HSE data. For the purpose of projecting YLL, all the individuals in any particular obesity category are assumed to have the average measurement for that category.

### Estimating Hazard Ratios by Age of Adult Life

To prepare the data for the Cox proportional hazards model, the following adjustments were made to the HALS data. Individuals with missing height, weight or waist values were removed from the dataset. Pregnant females were also excluded.

Cox proportional hazards models were estimated for non-smokers, using the following predictor variables: BMI, BMI^2^, WHtR, WHtR^2^, Age and Age^2^ plus certain combinations thereof. The inclusion of quadratic terms allows the effect of a predictor variable to change over the range of inputs.

Obesity measures might have a disproportionately large effect on mortality at very high levels and it is necessary to allow for this. SPSS 16.0 was used to fit the parameters to the model using the built in maximum likelihood estimation algorithm. This approach is explained in more detail in [26]. Interaction terms (BMI x Age, BMI^2^×Age etc.) were also tested and incorporated where the model fit was enhanced as a result.

The Cox proportional hazards model took the form:
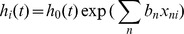
where:



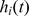
 = hazard (force of mortality) of *i^th^* individual at time t,



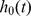
 = baseline hazard at time *t,*



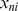

*are covariates,*



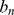

*are covariate coefficients*.

For choice of covariates, we tested a wide range of options including interaction terms involving combinations of two covariates (see Table 2). Not surprisingly, age was a common factor in all specifications of the model since age and mortality are so strongly linked.

**Table 2:** Cox proportional hazard model coefficients for BMI and WHtR.

Variable	BMI coefficients		WHtR coefficients	
Sex	Male	Female	Male	Female
*Age*	0.1725	0.1087	0.1886	0.1518
	(0.1125, 0.2324)*	(0.1018, 0.1156)*	(0.128, 0.2493)*	(0.1077, 0.196)*
*(Age)^2^*	−0.0005105	n.a.	n.a.	n.a.
	(−0.0009845, −0.0000365)*			
*BMI*	−0.1949	−0.1605	n.a.	n.a.
	(−0.3519, −0.03784)*	(−0.2795, −0.04143)		
*(BMI)^2^*	0.004038	0.00302	n.a.	n.a.
	(0.001299, 0.006777)*	(0.0009275, 0.005112)		
*WHtR*	n.a.	n.a.	n.a.	n.a.
*(WHtR)^2^*	n.a.	n.a.	11.43	7.439
			(5.038, 17.83)*	(2.469, 12.41)*
*(WHtR x Age)*	n.a.	n.a.	−0.1534	−0.09009
			(−0.2619, −0.04493)*	(−0.1726, −0.00762)*

*Coefficients passed the likelihood ratio test for inclusion into the final model.

*Note: “n.a.” means not applicable. Figures in brackets are 95% confidence intervals. Table excludes smokers.*

In this model, the log of the mortality rate is estimated as a function of age and obesity. This model assumes that the effect of the covariates (explanatory factors) is constant over time. In this case, the hazard rate can be interpreted as the force of mortality (or the instantaneous rate of mortality). It should be noted that this model cannot be used to assess the likely lifespan of a particular individual of a certain age and physiology as it does not allow for the likely change in the individual’s physiology over their lifetime (i.e. we assume that each individual’s BMI or WHtR remains constant over their lifetime).

### Estimating Expected Years of Life Lost

The 2006 interim life tables for the United Kingdom produced by the Office for National Statistics were used to provide the conditional probability of deaths for the total population [18]. The smoothed BMI distribution, together with the hazard ratios and the life tables, were used to produce estimates of YLL. Results based on WHtR were produced in a similar way. The steps involved in this method were the same as those used by other authors [21].

YLL for a person aged, say 30 years, in a particular BMI category relative to a person with optimum BMI (in this case 24 kg/m^2^ for males and 26 kg/m^2^ for females) is found by subtracting the expected age at death for that BMI category from the expected age at death in the optimum BMI category. Similarly YLL was estimated for WHtR by comparing to optimum values of WHtR (in this case 0.5 for males and 0.46 for females). The optimal measurements are found by comparing the life expectancies for differing WHtR/BMI measurements within the same age. Those measurements that produce the largest life expectancies are deemed to be the optimal ones.

## Results

### Mortality rates and obesity based on HALS

We repeated the work of Cox and Whichelow [6] to see if the relationships of BMI and WHtR with mortality had changed with the collection of ten years’ more HALS data.

**Figure 1:**
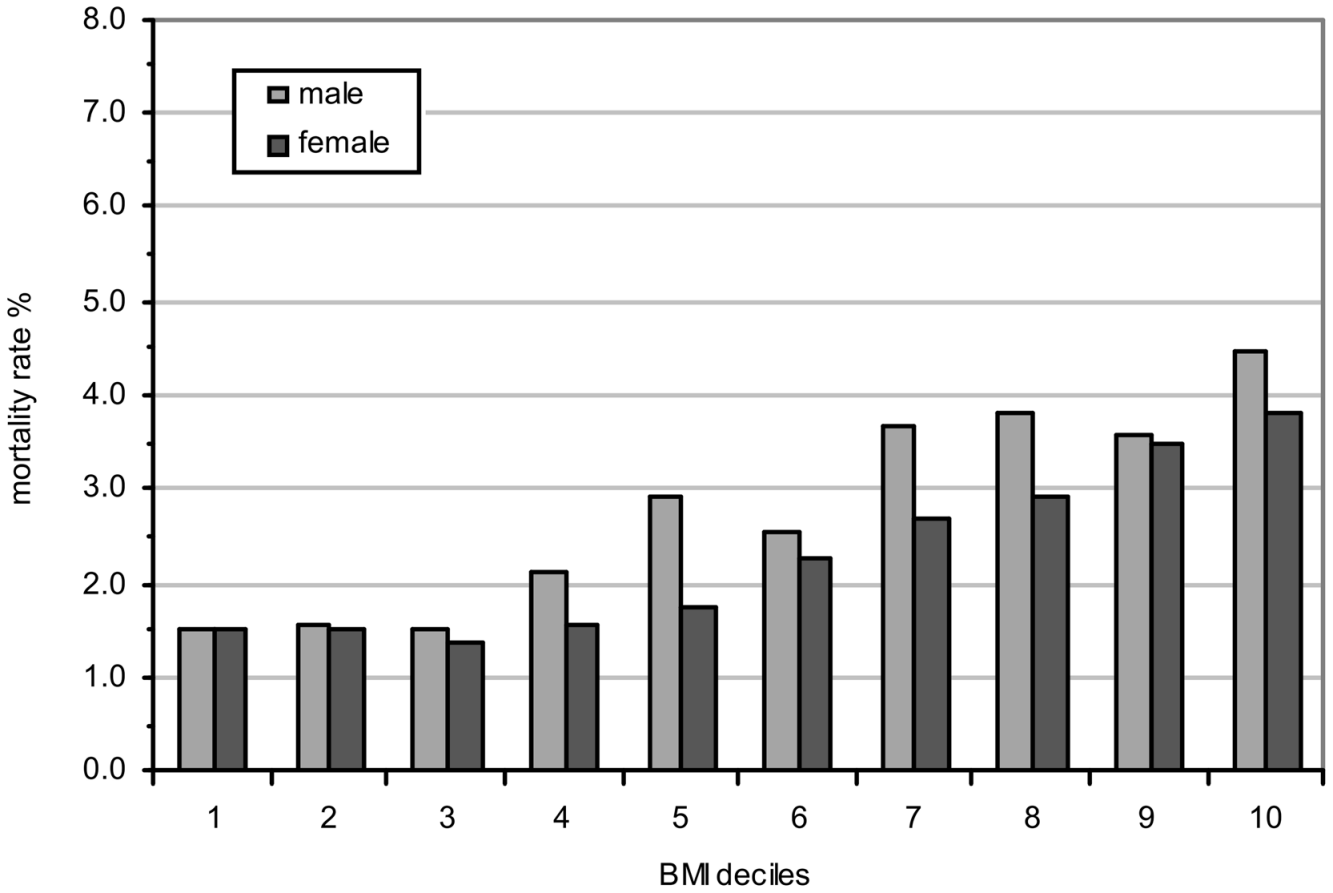
Mortality rate by BMI decile (source: HALS). A clear upward trend is apparent for both sexes and obesity tends to affect the mortality rates of males more than females. Logistic regression analysis of the probability of death versus BMI category confirms a statistically significant gender difference (p<0.01).

**Figure 2:**
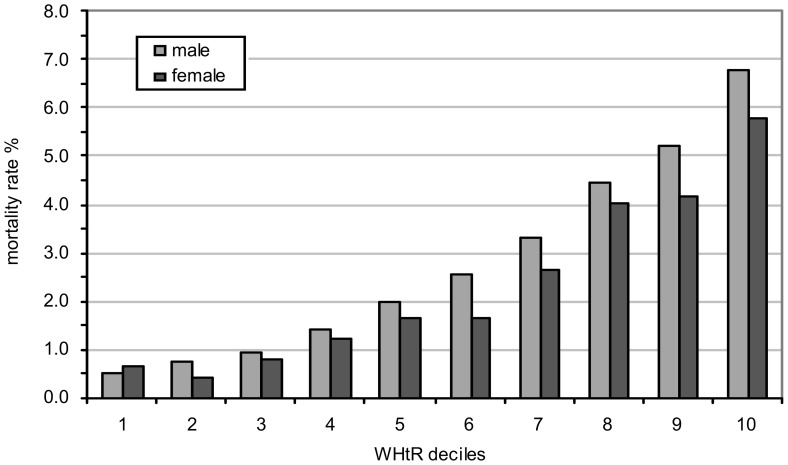
Mortality rate by WHtR decile (source: HALS). WHtR is a significantly better predictor of mortality than BMI for males and females (i.e. steeper mortality gradient across the deciles for WHtR). Use of regression analysis showed that the difference in slopes between mortality rate and BMI (Figure 1) and WHtR (Figure 2) was statistically different (p<0.01).

Figures 1 and 2 show the 20 year all-cause mortality rate by deciles (tenths) based on BMI and WHtR for males and females. The mortality rate is the number of deaths in a decile of BMI (or WHtR) divided by the number of people originally in that decile, expressed as a percentage.

For each measure, a clear upward trend is apparent for both sexes; obesity tends to affect the mortality rates of males more than females. Logistic regression analysis of the probability of death versus BMI or WHtR category confirms a statistically significant gender difference (p<0.01).

Further inspection of both Figures shows that WHtR is a more sensitive predictor of mortality than BMI for males and females (i.e. based on a steeper mortality gradient across the deciles for WHtR). Use of regression analysis showed that the difference in slopes between mortality rate and BMI (Figure 1) and WHtR (Figure 2) was statistically different (p<0.01). The mortality rate for males, based on BMI, increases from 1.5% to 4.5% across the deciles. In contrast, the mortality rate, based on WHtR, increases from 0.5% to just under 7%. Similarly for females, the mortality rate based on BMI increases from 1.5% to just under 4% whereas the mortality rate based on WHtR increases from 0.6% to just under 6% across the deciles. In addition, the relationship between mortality rate and WHtR deciles (Figure 2) has a smoother gradient than that for BMI (Figure 1), particularly for males.

In terms of the correlation between mortality rate and within decile median value of either measure, the results show that both BMI and WHtR perform well, but that the correlation between mortality rate and WHtR is higher than that between mortality rate and BMI for both genders. For males, we obtained a Pearson correlation coefficient of 0.98+/−0.07 where 0.07 is the standard error for WHtR and 0.91+/−0.15 for BMI. For females the equivalent results were 0.97+/−0.10 and 0.96+/−0.11.

Thus, the 20-year follow up data from HALS not only confirms the results from the 10-year data, but lends further support to the premise that WHtR is a superior predictor of mortality than BMI, particularly in the case of males [21].

### Fit of proportional hazards models

We used the Cox proportional hazards model to estimate the mortality rates of individuals at different ages. We fitted two stepwise regression models for males and females separately using SPSS: one model for BMI and the other for WHtR. We examined the change in the log likelihood to decide whether to include a variable or not based on standard criteria.

Table 2 shows the coefficients and 95% confidence intervals for each model. For the WHtR model the best combination of covariates was Age, (WHtR)^2^ and a composite variable (WHtR x Age) for both males and females. For the male BMI model, the inclusion of BMI, (BMI)^2^ and (Age)^2^ proved to be the best covariate combination. For the female BMI model, BMI and (BMI)^ 2^ did not significantly improve the model fit over age alone. Nevertheless, although the fitted parameters did not pass the inclusion test they did produce results that were consistent with past research [21] and so have been retained.

It would appear counter intuitive that, for both genders, the BMI coefficient is negative. However, the main relationship between BMI and mortality is modelled by the (BMI)^2^ parameter; the negative BMI coefficient has the effect of dampening the mortality rate increase, particularly for people at the lower BMI values.

On the basis of our analysis, we concluded that WHtR was better than BMI at predicting mortality, because it resulted in a better fit for both males and females and the results were more intuitive. We then used the predicted mortality rates from each model to estimate the YLL at different ages as described in the next section.

### YLL results according to different anthropometric indices

In this section we report the years of life lost (YLL) for different values of BMI and WHtR. Using the results of the Cox proportional hazards model, we converted predicted mortality rates at different ages and obesity values into life expectancies. We calculated YLL by subtracting the life expectancy of an individual having particular BMI or WHtR measurements from the life expectancy of a person of the same age with optimal measurements. For this purpose, we derived an optimal value of BMI of 24 for males (26 for females) and optimal value of WHtR of 0.5 for males (0.46 for females).

**Figure 3:**
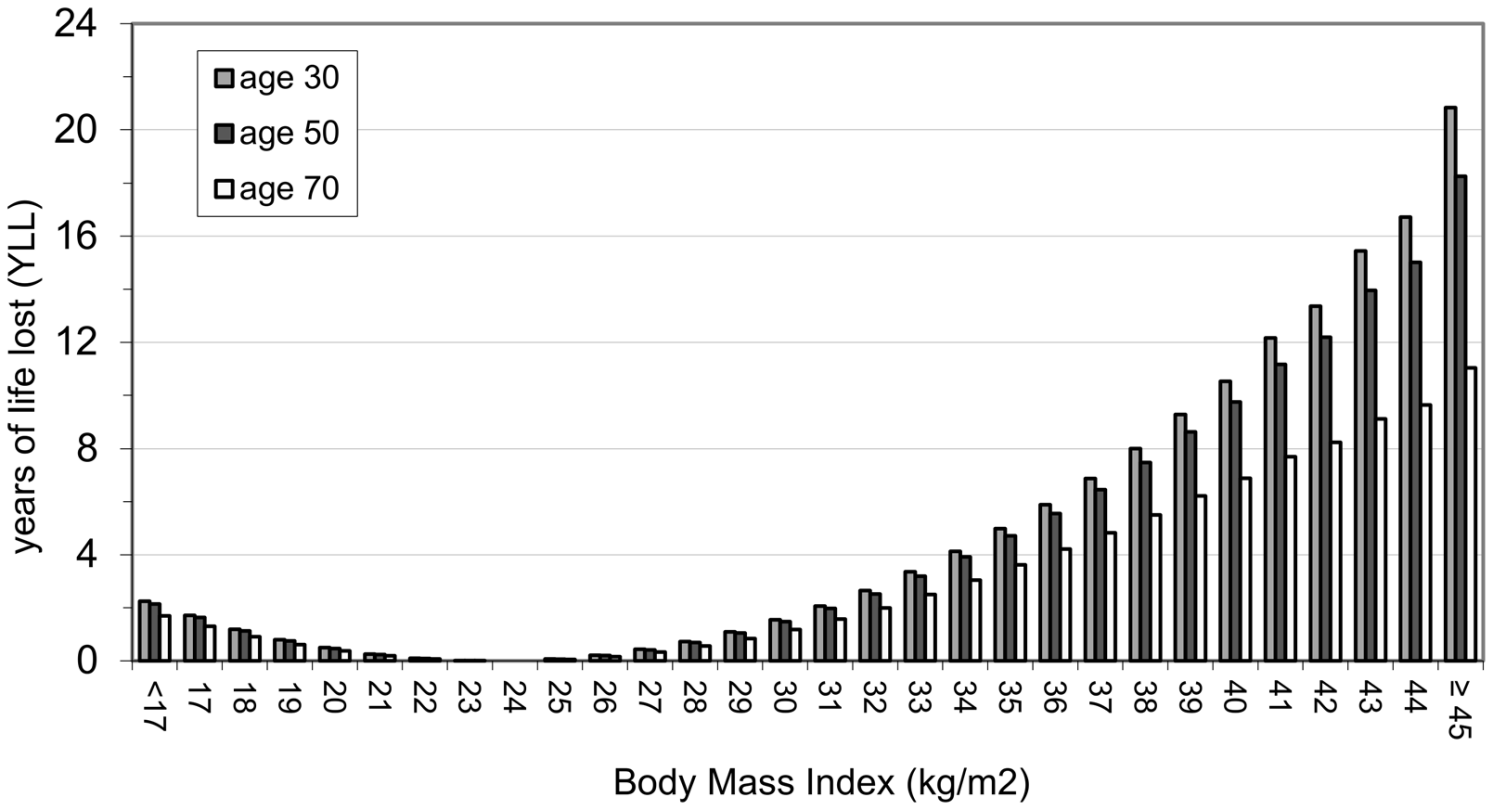
YLL relative to BMI 24 in male non-smokers. There is a J-shaped association between BMI and YLL at all three of the representative ages. The optimal YLL is at BMI 24 kg/m^2^ for males and YLL figures relate to this reference BMI value. The ‘accepted normal’ BMI category ranges from 18.5 to less than 25 kg/m^2^. Surprisingly, males have slight increased YLL in part of the ‘normal’ BMI category (ie BMI from 18.5 to to 22 kg/m^2^). Males have increased YLL compared with females in the ‘overweight’ category (ie BMI from 25 to 30 kg/m^2^) at all three of the representative ages.

**Figure 4:**
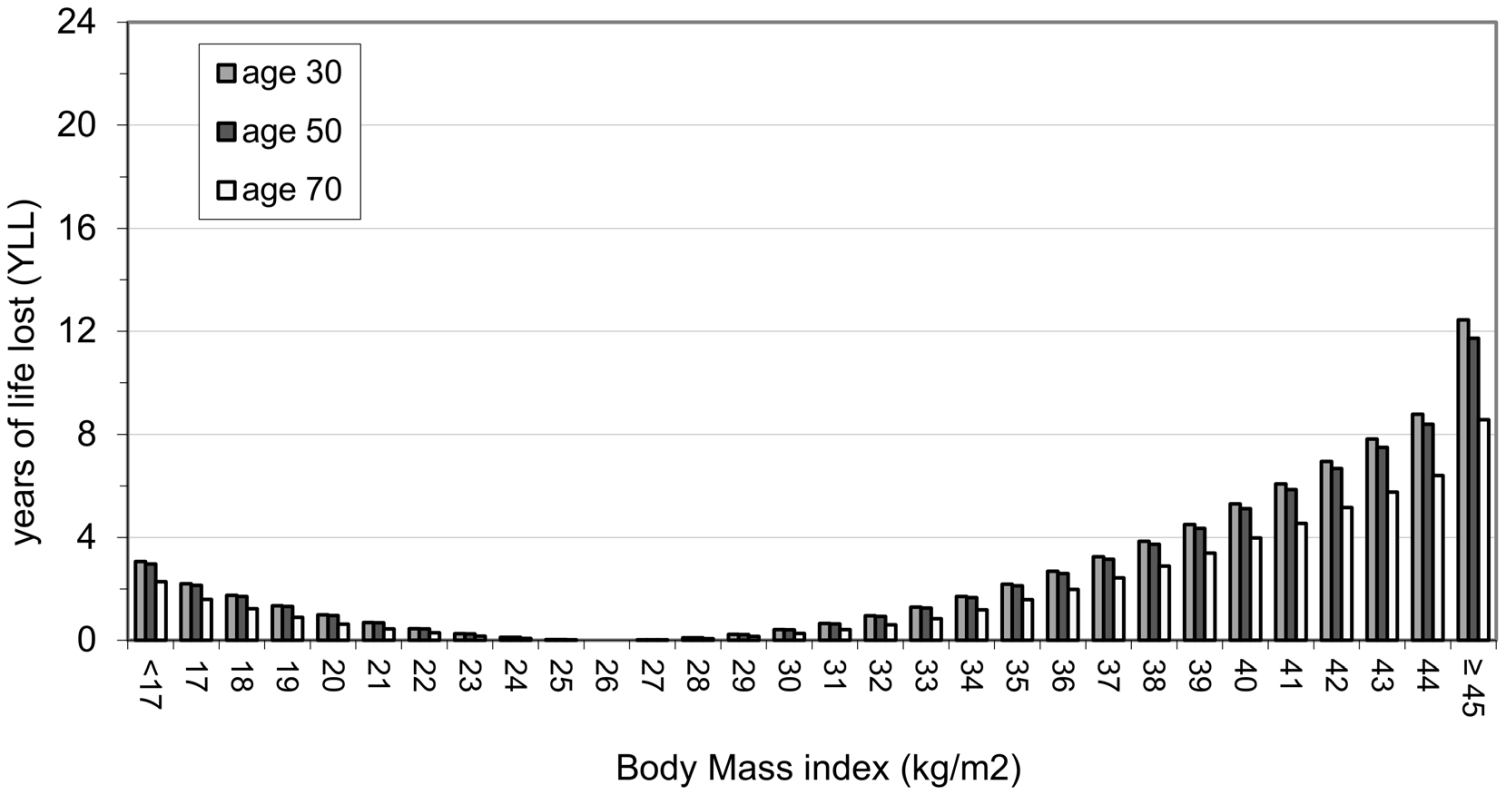
YLL relative to BMI 26 in female non-smokers. There is a J-shaped association between BMI and YLL at all three of the representative ages. The optimal YLL is at BMI 26 kg/m^2^ for females and YLL figures relate to this reference BMI value. The ‘accepted normal’ BMI category ranges from 18.5 to less than 25 kg/m^2^. Surprisingly, females have slight increased YLL in part of the ‘normal’ BMI category (ie BMI from 18.5 to 24 kg/m^2^).

**Figure 5:**
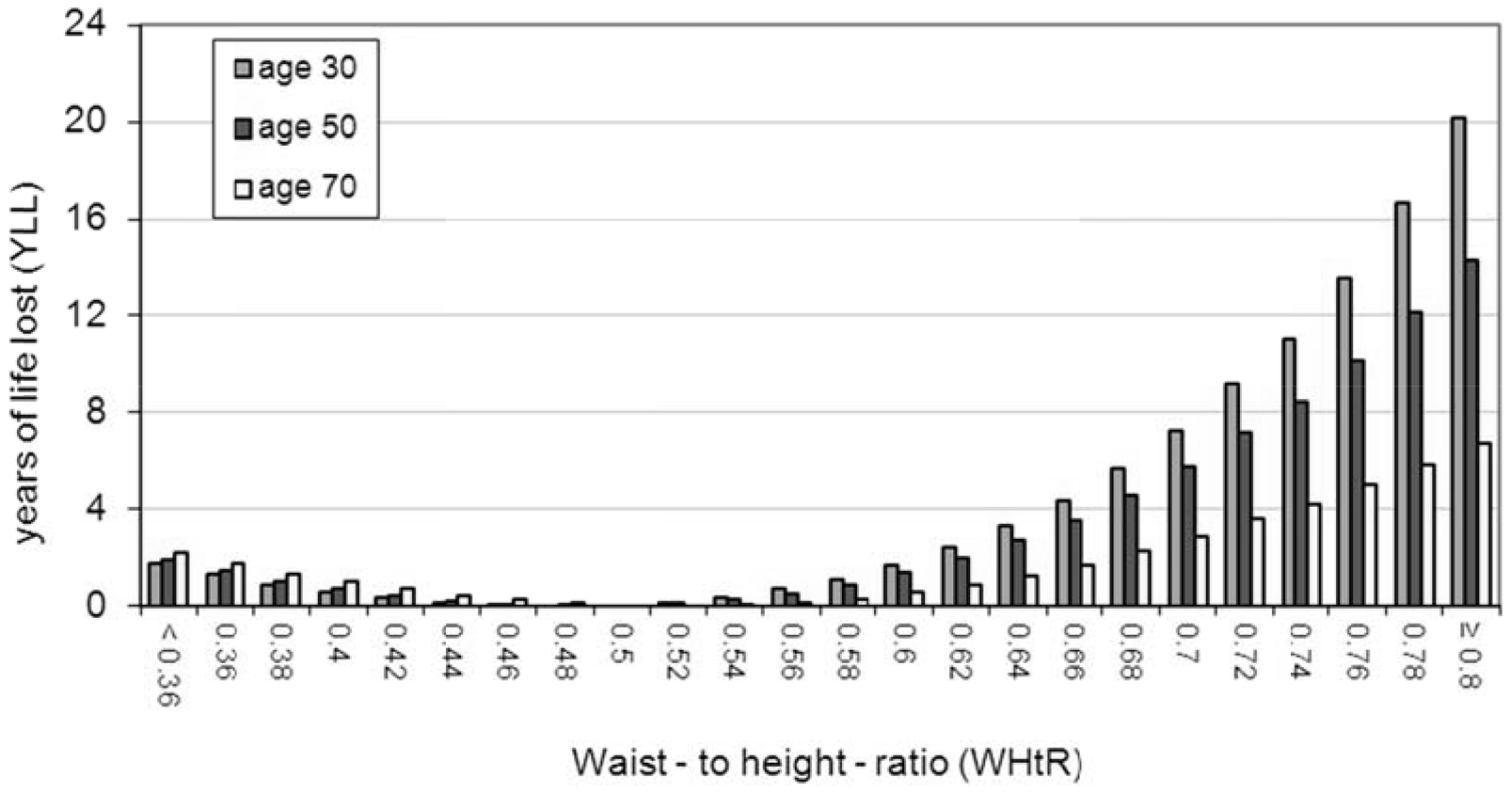
YLL relative to WHtR 0.5 in male non-smokers. There is a J-shaped association between WHtR and YLL at all three of the representative ages. The optimal YLL is at WHtR 0.5 and YLL figures relate to this reference value. There is minimal increased mortality risk in the ‘OK’ range of WHtR i.e from 0.4 to 0.5. At the lower two representative ages, males have an increased risk of mortality if they are in the ‘Consider Action’ (WHtR 0.5 to 0.6) range. YLL increases markedly after WHtR 0.6 (the ‘Take Action’ category) at all three of the representative ages.

**Figure 6:**
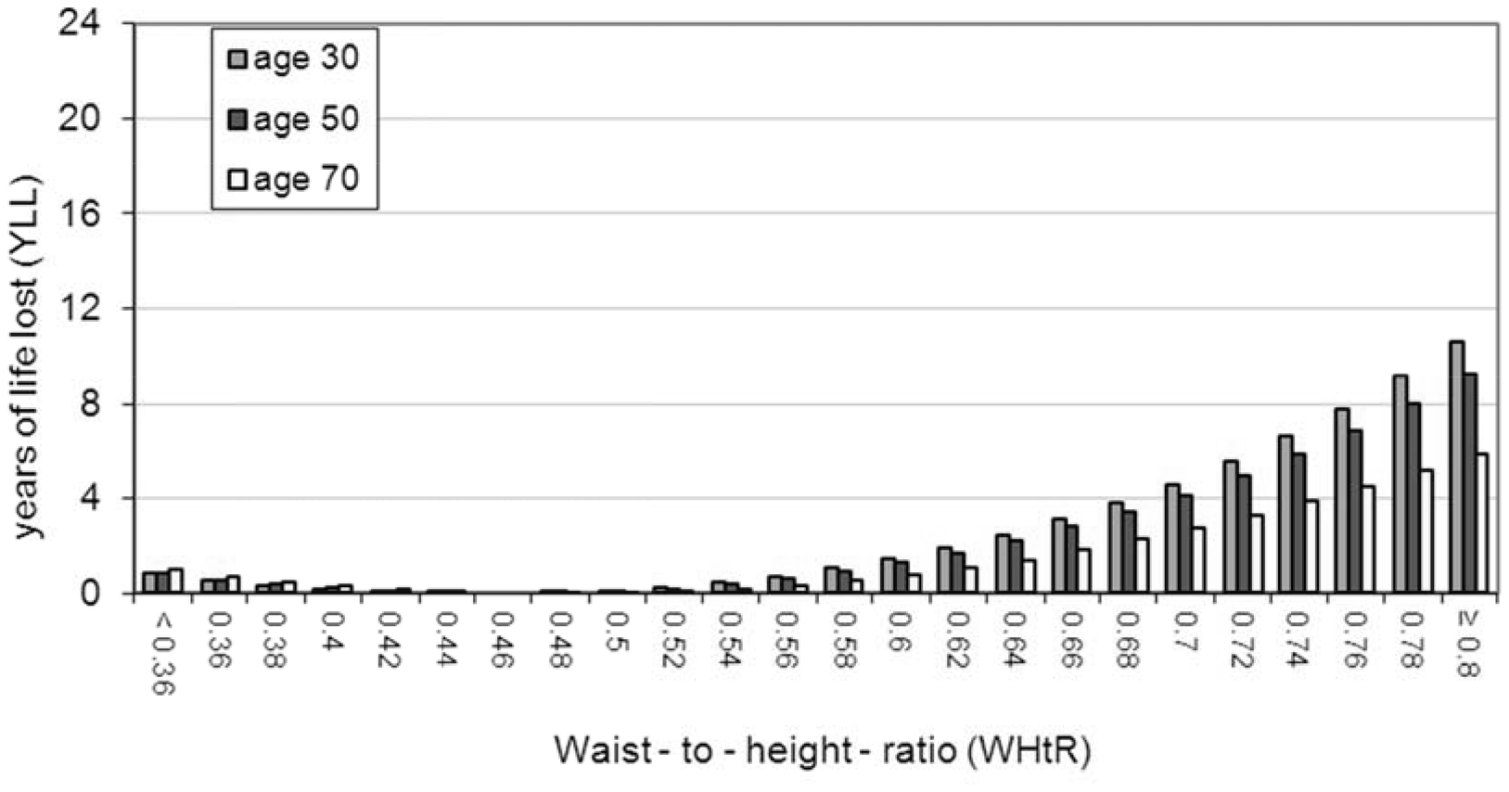
YLL relative to WHtR 0.46 in female non-smokers. There is a J-shaped association between WHtR and YLL at all three of the representative ages. The optimal YLL is at WHtR 0.46 and YLL figures relate to this reference value. There is minimal increased mortality risk in the ‘OK’ range of WHtR i.e from 0.4 to 0.5. At the lower two representative ages, females have an increased risk of mortality if they are in the ‘Consider Action’ (WHtR 0.5 to 0.6) range. YLL increases markedly after WHtR 0.6 (the ‘Take Action’ category) at all three of the representative ages.

A summary of the results is shown in Table 3 where results for both males and females are given at the representative ages of 30, 50 and 70 years. For each category, the table shows the percentage of the whole population above the stated value of the anthropometric index and the expected future years of life lost (YLL) at the given age. Figures 3 to 6 show the results graphically for male and female non-smokers for the three representative ages. The results assume that each individual stays with their particular anthropometric index throughout the rest of their life.

**Table 3:** Summary of YLL results based on BMI and WHtR for males and females at three representative ages: 30, 50 and 70 y.

Age 30 years	male				female			
BMI value(kg/m^2^)	25	30	35	40	25	30	35	40
% above BMI value^1^	59.3%	21.2%	5.3%	0.8%	48.4%	19.6%	6.8%	1.9%
YLL	0.1	1.6	5.0	10.5	0	0.4	2.2	5.3
WHtR	0.5	0.6	0.7	≥0.8	0.5	0.6	0.7	≥0.8
% above WHtR value	62.1%	11.9%	1.3%	0.2%	41.7%	8.9%	1.6%	0.3%
YLL	0	1.7	7.2	20.2	0.1	1.4	4.6	10.6
**Age 50 years**								
BMI value(kg/m^2^)	25	30	35	40	25	30	35	40
% above BMI value^1^	75.3%	32.4%	8.0%	1.4%	63.3%	29.2%	10.9%	3.0%
YLL	0.1	1.5	4.7	9.7	0	0.4	2.1	5.1
WHtR	0.5	0.6	0.7	≥0.8	0.5	0.6	0.7	≥0.8
% above WHtR value	85.1%	27.1%	3.1%	0.5%	65.9%	20.5%	4.0%	0.7%
YLL	0	1.4	5.8	14.3	0.1	1.3	4.1	9.2
**Age 70 years**								
BMI value(kg/m^2^)	25	30	35	40	25	30	35	40
% above BMI value^1^	74.8%	31.7%	7.6%	1.4%	69.6%	33.8%	12.1%	3.1%
YLL	0.1	1.2	3.6	6.9	0	0.3	1.6	4.0
WHtR	0.5	0.6	0.7	≥0.8	0.5	0.6	0.7	≥0.8
% above WHtR value	93.0%	42.4%	5.4%	0.7%	80.8%	33.7%	7.2%	1.4%
YLL	0	0.5	2.9	6.7	0	0.8	2.7	5.9

1 HSE percentages for whole population.

#### General results relevant to both BMI and WHtR (Table 3 and Figures 3 to 6)

There is a clear J-shaped association between the two anthropometric indices and YLL (see Figures 3 to 6). Males and females at older ages lose fewer years from obesity (total or central) than those at younger ages (see Table 3). For example a 30 year old female non-smoker with WHtR 0.7 has YLL of 4.6 whereas her 70 year old counterpart has YLL of 2.7. The simple reason for this is that older people have fewer years to lose relative to normal life expectancy since life expectancy decreases with age attained.

For both BMI and WHtR, there is more variation in YLL between the three age groups for males than for females (eg range of 20.2 to 6.7 YLL for males at highest WHtR compared to 10.6 to 5.9 YLL for females at highest WHtR - see Table 3).

YLL would be lower if the group of lives included smokers (results not shown). This is because smokers have a lower life expectancy than non-smokers. The group as a whole would therefore have a lower life expectancy compared to a group containing only non-smokers. This combined group would therefore have fewer years of life to lose, on average, from the effects of obesity. The effect of including smokers would therefore be to dampen the YLL results shown in Table 3.

#### Results relevant to BMI (Table 3 and Figures 3 and 4)

The accepted BMI ‘normal’ range (i.e for optimal health) is from 18.5 to less than 25 [14]. The optimal YLL derived in this study is at BMI 24 for males and 26 for females; the YLL values in Figures 3 and 4 relate to these reference BMI values. Surprisingly, both males and females have some increased YLL in part of the ‘normal’ BMI category. The region where the increased YLL occurs tends to be between 18.5 and 22.

Males have increased YLL compared with females in the ‘overweight’ category (ie BMI from 25 to 30 kg/m^2^) at all three of the representative ages (Figs. 3 and 4). YLL increases markedly after BMI 30 kg/m^2^ (the ‘obese’ category) in both sexes. However, obese males have greater YLL than obese females at all three of the representative ages. For example, a 30 year old male with a BMI of 35 is expected to lose 5 years compared with 2.2 years for the corresponding 30 year old female (see Table 3).

#### Results relevant to WHtR (Table 3 and Figures 5 and 6)

One of the authors has previously proposed that the WHtR range of 0.4 to 0.5 should be considered as ‘OK’ [25]. Many other researchers in this field use WHtR 0.5 as a boundary value [8] with increased risk starting above 0.5 (‘Consider Action’ category) and substantially increased risk starting at WHtR over 0.6 - the ‘Take Action’ category [25].

In our study, the minimal value of YLL for males was at WHtR 0.5 and for females was at 0.46; the YLL values in Figures 5 and 6 relate to these reference values of WHtR. There is minimal increased mortality risk for either sex in the ‘OK’ range of WHtR i.e from 0.4 to 0.5.

Both sexes, certainly at the lower two representative ages, have an increased risk of mortality if they are in the ‘Consider Action’ (WHtR 0.5 to 0.6) range. YLL increases markedly after WHtR 0.6 in both sexes (the ‘Take Action’ category). However, males have greater YLL than corresponding females at all three of the ages. For example, a 30 year old male with a WHtR 0.7 is expected to lose 7.2 years compared with 4.6 years for the equivalent female (see Table 3).

## Discussion

### BMI results

#### Comparison between Britain and USA

The J-shaped association between BMI and YLL is similar to those found by others for the white US population when BMI is plotted against YLL or increased mortality risk [21], [26], [27] and to that found in a collaborative analysis of 57 prospective studies in western Europe and the US [28].

However, our results for Britain suggest a steeper growth in YLL as BMI increases and smaller YLL from BMI categories in the unhealthy low range (BMI less than 20). As in the US [21], males were observed to have higher YLL for a specific BMI than females and ‘overweight’ was not found to be a serious health issue - with US females only showing signs of increased YLL in the ‘overweight’ BMI category of 28. Recently, a systematic review has been published of reported hazard ratios for all cause mortality and BMI. In a sample size of nearly 3 million US individuals, overweight was actually associated with a significant lower all cause mortality than normal weight. Only Grade 2 and 3 obesity were associated with higher all-cause mortality [29].

US researchers also considered YLL and mortality separately for black males and females [26] and found a much flatter relationship between YLL and BMI for the white population. Therefore, if the US studies had combined the results for the white and black populations, their overall results would have been flatter. By implication, it is possible that the results demonstrated in our study might have been diluted by the effect of combining ethnic groupings. There is the possibility that significant differences exist between different ethnicities within Britain. However, there were insufficient data on ethnic mix within the data to examine this possibility further.

Fontaine et al [21] controlled for smoking as a potential confounder of BMI and YLL, whereas we chose to present results for non-smokers alone because of this acknowledged confounding influence and because of concern about the suitability of the data.

### Waist-to-height ratio results

Although several prospective studies have shown that WHtR is a better predictor of morbidity than BMI e.g. [30] [31] [32], we are aware of only one study which has looked at all cause mortality in relation to BMI and WHtR [6] and none which have calculated YLL for both anthropometric indices.

The 20-year follow-up results from the HALS data presented here are consistent with those previously calculated using the 10-year follow-up HALS data [6]. The previous study carried out logistic regression analysis, with adjustment for age and smoking, in 2,184 men and 2,730 women aged 30–79 years. This showed that, whereas BMI did not significantly predict death from all causes of cardiovascular death, WHtR was a significant predictor (P<0.01) of both death from all causes and from cardiovascular causes. Our results, based on nearly 2,000 deaths from the same cohort, support and strengthen the earlier findings.

Overall, our results on WHtR suggest that YLL increases dramatically from categories in excess of WHtR 0.52 for both males and females (see Figures 5 and 6). A recent systematic review collated global studies using specificity and sensitivity analyses in cross-sectional studies to estimate prediction of cardiometabolic risk for waist-to-height ratio. Based on optimal specificity and sensitivity, the review has suggested a boundary value of WHtR 0.5 [8]. Many authors have used this simple WHtR boundary value to indicate first level of risk - not least because it converts to the simple message “Keep your waist circumference to less than half your height”. Substantially increased risk has been suggested to start at WHtR 0.6 but this value has only been set pragmatically. Similar pragmatic reasoning has been used for the boundary value of WHtR 0.4 [25]. Our results using YLL not only lend support to all these proposed boundary values but they also help to quantify the effects in terms of reduced life expectancy. The results from this method of quantification could be used to try to persuade obese people to reduce the fat around their waist.

### Limitations of our study

**Use of HALS data.** The following points should be noted about our analysis of the prospective HALS data:

- Some research [33] suggests that participants who die early may distort the results because terminal illness is associated with low body fat. Previous research on HALS data [6] suggests that this effect is marginal. No account in our analysis was taken of other kinds of risk factors (such as diabetes or high blood pressure) in estimating between the predictor variables and mortality. This is, therefore, potentially an area for further research.
- The sample size at very high obesity levels is small in this dataset. The consequence is that there will be more uncertainty around the results obtained for the proportional hazard ratios at the highest levels of obesity.
- Unlike the US study on which our methodology is based [21], we took no account of ethnicity. This was because our British dataset was too sparse to distinguish between ethnic groupings. The US findings were that the influence of obesity on mortality was much greater in the white compared with the black population. This was discussed in more detail above.
- We are aware that, during the period while we have been carrying out our research, another HALS dataset has been released (which extends the follow-up period to 2009). We intend to carry out a similar analysis on the later dataset in due course.

**Calculation of YLL.** The following points should be noted about our calculation of YLL:

- The proportional hazards model assumes that the coefficients are constant over time. To check that this assumption was met in practice, we plotted Shoenfield residuals against survival time for each independent variable. We found them to be independent of time and so concluded that this assumption was acceptable (as it was in the US study [21]).
- The relatively small sample size of individuals in the severest obesity categories means that as both BMI and WHtR increase, the derived hazard ratios become less precise. These have a large influence on the YLL figures. It would be helpful to calculate confidence intervals. However, as noted in [21], this is very difficult to do in practice since the data used in the study come from 3 different sources. Our results support the results of other US studies [21], [26], [27]. To clarify this uncertainty it would be helpful if a similar analysis could be applied to a much larger dataset containing a higher proportion of overweight and obese individuals.
- The YLL calculations assume that BMI or WHtR remains constant over the individual’s future lifetime. Therefore, the YLL results described earlier are always a result of comparing an individual in (and remaining in) a certain BMI (or WHtR) category with an individual in (and remaining in) the optimum BMI (or WHtR) category. In extending this research it would be preferable to use longitudinal data in which there would be included continuous measures of body fat at every age, although clearly this would have implications in terms of both cost and time needed to do the research.

### Strengths of our approach

- We have used 20-year prospective data from the HALS survey. Although others have used data on many more individuals from NHANES, their prospective data is only over a period of 5 years [34].
- We have used British data to quantify and compare YLL using BMI and WHtR. To the best of our knowledge we are the first to use WHtR to quantify YLL.

### Generalisability and policy implications

Growth in Britain of the obese and morbidly obese categories of the population has been substantial since the start of the HALS investigation (1985). A similar study repeated now would have a larger sample of such individuals but it would take time before a suitable period of follow-up had elapsed to provide reliable results.

This paper covers a topic that is important to the planning of health care, social policy and insurance in Britain. Through further analysis of the HALS prospective data [17], our study suggests that the mortality risk associated with obesity in Britain is similar to that found in US studies. It finds that a 30 year-old male non-smoker with a BMI of 40 is expected to live 10.5 years fewer than a 30 year-old male with a BMI of 24. The corresponding figure for a 30 year old female (BMI 40 compared to BMI 26) is 5.3 years. These examples reflect the overall results which suggest that obesity is more of a risk for males than females.

Our research also supports the premise that WHtR is a better predictive risk measure of mortality than BMI [7], [9]. We have been able to quantify the YLL for different values of WHtR at three representative ages. For example, we find that a 30 year-old male non-smoker with a WHtR of 0.7 is expected to live 7.2 years fewer than a 30 year-old male with a WHtR of 0.5. The corresponding figure for a 30 year old female (WHtR 0.7 compared to WHtR 0.46) is 4.6 years.

The evidence presented here suggests that government policy and future research should therefore place more emphasis on WHtR as a screening tool. Current UK policy tends to be restricted to BMI and waist circumference [35], [36]. We argue that focusing on WHtR will identify those with central obesity and will focus resources on those most at risk.

Although waist circumference is a good proxy for central obesity, there are problems with setting cut-off values that can be used for all ethnic groups [25] and for children. WHtR has the advantage that, by making allowance for height, the same cut-off, or boundary value, (0.5), can be used for everyone [9].

Other authors have suggested the use of A Body Shape Index (ABSI) as a way to quantify abdominal obesity [34]. Interestingly, changes in ABSI have also been calculated between two HALS examinations seven years apart and shown that greater mortality risk was shown in those people with initial high ABSI who had a rising ABSI between examinations [37]. However the calculation of ABSI is based on waist circumference relative to height and BMI. We believe it would be much too complicated to calculate ABSI for practical purposes. Further, a comparison of various surrogate obesity indicators as predictors of CVD mortality in four European populations found the prediction with WHtR to be stronger than that with ABSI [38].

Other anthropometric indices, such as saggital abdominal diameter [39], could be even more accurate than WHtR in predicting mortality risk, but we believe that the logistics of measurement are of great importance and a simple index such as waist-to-height ratio has great practical advantages:

- Our YLL data support the pragmatically determined boundary values for WHtR, which can be used to promote very simple public health messages. Thus, WHtR between 0.4 and 0.5 is OK; WHtR between 0.5 and 0.6 signifies ‘Consider Action’; and WHtR above 0.6 indicates ‘Take Action’ [25].
- This first boundary value of WHtR 0.5 gives rise to the simple message “Keep your waist circumference to less than half your height”. Our research now quantifies the effects of not doing so. The promotion of this simple message could be powerful in persuading people to consider, or take, action if their WHtR is inappropriate. [9]. If a tape measure is not available, a piece of string could suffice!

This paper is a response to the concern that, worldwide, the prevalence of obesity has been increasing over the last few decades. It is known that obesity can lead to several diseases, in particular cardiovascular diseases, diabetes, and various cancers. It has been predicted that there will be 11 million more obese adults in the UK and 65 million more obese adults in the US by 2030. By then, it is estimated that in the UK and US combined, there will be an additional 6–8.5 million cases of diabetes, 5.7–7.3 million cases of heart disease and stroke and 0.5 to 0.7 million additional cases of cancer. In total, taking UK and US together, 26–55 million quality-adjusted life years will have been lost. The total medical costs associated with these chronic diseases are estimated to increase by £1.9–2.0 billion per year in the UK and by $48–66 billion per year in the US by 2030. Therefore, there are considerable economic benefits to reducing the levels of obesity worldwide [10].

On present trends, health care providers will find themselves treating more people for diseases caused by excess body fat. Our study has focused on the relationship between obesity and mortality. Many of the diseases associated with obesity, such as diabetes, are also associated with factors such as smoking habits or genetic pre-disposition. Thus, it is difficult to separate out the influences of each with any certainty in order to estimate morbidity as well as mortality reductions; however, death is likely to have been preceded by a period of higher health care consumption especially where the cause of death is from chronic disease. In this regard, further work is needed around rates of obesity-associated diseases and their relationship to age, gender and other risk factors. Nevertheless, the research presented here emphasizes how important it is for the government to promote healthy lifestyles in order to avoid premature death (i.e. YLL).
